# Child and adolescent mental health service provision and research during the Covid-19 pandemic: challenges, opportunities, and a call for submissions

**DOI:** 10.1186/s13034-020-00324-8

**Published:** 2020-05-11

**Authors:** Andreas Witt, Anna Ordóñez, Andrés Martin, Benedetto Vitiello, Jörg M. Fegert

**Affiliations:** 1 Department of Child and Adolescent Psychiatry and Psychotherapy, University of Ulm, Ulm, Germany; 2 National Institute of Mental Health, Bethesda, MD USA; 3 Child Study Center, Yale School of Medicine, New Haven, CT USA; 4 Division of Child Neurology and Psychiatry, Department of Public Health and Pediatric Sciences, Regina Margherita Pediatric Hospital, University of Turin, Turin, Italy

*Although the virus has mercifully had a small direct impact on children and those underage, it has shown no such mercies on the families and extended community of caretakers supporting children. Even if this is not a ‘childhood pandemic’, it is very much a family and society pandemic. It is clear that the reverberations of this time will be enduring, and that our work will be more needed than ever.*

– *Message from the IACAPAP President and Executive Committee, April 15, 2020*

The first human cases of Covid-19 were identified in December 2019 [1]. Since then, the virus has spread around the world and, by March 2020, most European countries as well as the US had started to report sharp increases in the number of cases [2]. As we write this editorial in late April, more than 2 million people have been infected around the globe and over 140,000 people have died [3]. Worldwide, researchers are on the hunt for a vaccine, but initial results are not expected for several months. Meanwhile, the fight against the virus continues with strategies to mitigate its consequences and to protect high-risk groups in the population. In general, measures of hygiene and distancing strategies now being used in most countries are aimed at slowing down the infection rate—at flattening the curve in order to avoid the collapse of overwhelmed health care systems.

We prefer the term ‘physical distancing’ over the commonly used ‘social distancing’. We are not the only ones [4], and our rationale is evident: we are social beings, and social connection is as necessary to our physiology as is nourishment. Many of us are fortunate to maintain physical distance or complete isolation while remaining virtually connected. But many across the globe don’t have access to the internet or to electronic devices through which to communicate with each other. We don’t take for granted the e-means through which we are spreading this message and reaching our community of peers. There remains much to be done to make virtual connections available for all, and especially for those in highest need. Indeed, virtual connectivity and the challenge to reach communities in need are at the core of our message—and of our entreaty to readers.

Measures of physical distancing include general ‘shutdowns’. This means that most businesses, schools and universities are closed, countries have imposed travel restrictions, people are encouraged—in some cases mandated—to stay at home, and contact restrictions and even curfews have been enforced. The Covid-19 pandemic is profoundly impacting all of our lives. Restrictive measures impose a complete change to our psychosocial environment.

Apart from the general public, the medical sector has been particularly affected by the crisis. In some countries the entire medical system has been reorganized. Medical systems have taken steps to rapidly increase their intensive care bed capacity by postponing elective procedures whenever possible, or by building ‘pop-up’ hospitals into which to decant non-critically ill patients. Retired doctors and nurses have returned to service and students of medical and nursing schools have volunteered in ‘drive-through’ corona testing stations. Students help out on the wards or at specific medical facilities to ensure basic care for all patients [5]. Some medical and nursing schools have graduated their seniors months ahead of schedule in order to meet the overwhelming demand for health care providers.

As the medical system understandably prioritizes care for the sickest patients and those at imminent risk of death, other areas, including the mental health sector, have received less focused attention [6]. Some forms of high-risk treatment, like day treatment units in child and adolescent psychiatry have closed, as have schools and aftercare programs. Strategies such as telehealth have increased rapidly, and mental health professionals have become creative in order to continue serving their patients. The pandemic has accelerated uptake of telepsychiatry in ways that early adopters of virtual strategies could not have dreamed of. Paradoxically, the pandemic has been a blessing for telepsychiatry—and may not be the only silver lining to an otherwise sobering public health crisis.

Different types of outpatient treatment are now delivered routinely through telephone or secure video platforms. Most institutions have installed pre-screenings to adequately triage health concerns and minimize contact risk at physical locations as much as possible. Inpatient treatment has been maintained in most places, although with major changes to usual practices (as but one example, ‘milieu-based’ services that are core to many inpatient units are challenging, though not impossible, to conduct under physical distancing). Screening and quarantine conditions have been instituted for patients in urgent need of inpatient care, but universal testing is not yet available in most countries, further compounding the challenges to admit children into congregate care settings. Visits to the wards have been massively limited or entirely eliminated, distancing rules maintained by staff wearing masks and other personal protection equipment. Overall, the nature of care and the human contact at its center have all changed dramatically and at dizzying speed. Adaptation and flexibility have become the name of the game.

From a developmental perspective, children, adolescents and young adults suffer increasingly from prolonged and massively restricted social contacts. Families are forced to re-organize and cope with new situations, such as quarantine and physical distancing. School closures have led to distance-learning and home-schooling—not to mention challenges to food distribution: school is the place where many children around the globe get a consistent daily caloric intake. School-based nutrition is a reality in both developing and developed countries [7–9]. Parents are experiencing increased pressure to work from home, to keep jobs and businesses running, as well as to support, educate and comfort their children at the same time. Many families don’t have the ability to isolate or restrict physically, with economic realities superseding public health prevention efforts. Additionally, parents have the role of explaining the pandemic and its consequences to their children, and to manage and contain the fear and anxiety that accompanies everyone in the family during these uncertain times. Under this panoply of acute adversity, children may be at higher risk than in normal times for abuse or neglect, and with protective care agencies also working under extreme and strained conditions, recognition and intervention may be delayed.

We know from prior epidemics that there can be linkages between anxiety and depression and viral diseases such as Influenza A (H1N1) [10]. We anticipate the current crisis will have a similar impact on the mental health of children and adolescents. In a recent review, Brook and colleagues [11] examined the psychological impact of quarantine and how to reduce its accompanying negative psychological effects, including posttraumatic stress, confusion and anger. Boredom and frustration may predict family stress and conflicts. The uncertain duration of the quarantine measures, the need for families to simultaneously work (or manage the stressors of employment loss), to provide childcare and to support distance-learning efforts, as well as to manage the impact of disease and loss of life in their families and communities may lead to long-lasting effects, many of them related to mental health and psychopathology.

Low-income families are particularly vulnerable, and there may be a widening gulf across economic, educational and social divides. The effects of poverty may be magnified, with a disproportionate toll on minority, marginalized, or already underserved communities. There is reason for concern that suicide rates may increase during the crisis as a consequence of distancing, quarantine and isolation [12]. Additionally, there is evidence from the major economic recession of the last decade that child maltreatment and intimate partner violence increases during such periods [13–15]. The omnipresent reports about death rates and people dying without relatives able to say goodbye evoke additional health-related fears that will affect persons in different ways. Against this sobering backdrop, the burden on children and adolescents, especially those already suffering from mental health problems, is expected to endure long after the pandemic is over.

And yet, we know that times of crisis and disruption offer opportunities for resilience, growth and extraordinary development [16–18]. It is in this spirit that Child and Adolescent Psychiatry and Mental Health (CAPMH) seeks submissions pertaining to child and adolescent mental health related to the Covid-19 pandemic. We are especially interested in empirical work on clinical service delivery or educational initiatives, and in any work that offers international collaborations or innovative solutions. We welcome submissions of a quantitative, qualitative, or mixed-methods nature. Our aim is to produce a curated issue of open-access resources available free of charge to our global readership. We encourage inquiries on potential submissions, and interested readers can submit their queries to the special issue section editors by contacting the corresponding author. We are partnering in this effort with the International Association of Child and Adolescent Psychiatry and Allied Professionals (IACAPAP), one of our parent organizations. IACAPAP has already collected a series of online resources from around the globe related to the Covid-19 pandemic. We encourage readers to make use of these materials and to suggest additional ones from their respective countries by clicking here.

This pandemic should redouble our commitment to children and families and to underserved communities all around the globe. It should also be a powerful reminder of the fact that we are not alone: we have each other. We hope that this message and the resources included in it may provide not only solace and comfort, but concrete tools with which to be better prepared as you go forward to do what you have committed your professional lives to do: to take care of the children and families we are privileged to serve.

## Data Availability

Not applicable.

## References

[1] World Health Organization (WHO). Q&A on coronaviruses (COVID-19). World Health Organization. 2020. https://www.who.int/news-room/q-a-detail/q-a-coronaviruses. Accessed 22 Apr 2020.

[2] Johns Hopkins University & Medicine. Cumulative cases. 2020. https://coronavirus.jhu.edu/data/cumulative-cases. Accessed 22 Apr 2020.

[3] Johns Hopkins University & Medicine. COVID-19 dashboard by the Center for Systems Science and Engineering (CSSE) at Johns Hopkins University (JHU). 2020. https://coronavirus.jhu.edu/map.html. Accessed 22 Apr 2020.

[4] Kort J. Practice physical distancing, not social distancing: how to cope with the coronavirus quarantine. Psychology Today 2020; 2020. https://www.psychologytoday.com/us/blog/understanding-the-erotic-code/202003/practice-physical-distancing-not-social-distancing. Accessed 22 Apr 2020.

[5] Schulze U, Fegert JM. Covid 19 and its impact on child and adolescent psychiatry—a German and personal perspective. Ir J Psychol Med. **(submitted)**. 10.1017/ipm.2020.43 PMC7411433 32404219

[6] Fegert JM, Kehoe LA, Vitiello B, Karwautz A, Eliez S, Raynaud J-P, et al. COVID-19: services must remain active, we must communicate with networking partners and avoid further closure of psychiatric units. 2020. https://www.escap.eu/index/coronavirus-and-mental-health/maintain-contactwith-patients-and-their-families-and-prevent-closure-of-services. Accessed 22 Apr 2020.

[7] Herforth A, Arimond M, Álvarez-Sánchez C, Coates J, Christianson K, Muehlhoff E (2019). A global review of food-based dietary guidelines. Adv Nutr.

[8] Oostindjer M, Aschemann-Witzel J, Wang Q, Skuland SE, Egelandsdal B (2017). Are school meals a viable and sustainable tool to improve the healthiness and sustainability of children’s diet and food consumption? A cross-national comparative perspective. Crit Rev Food Sci Nutr.

[9] Sabinsky MS, Toft U, Sommer HM, Tetens I (2019). Effect of implementing school meals compared with packed lunches on quality of dietary intake among children aged 7–13 years. J Nutr Sci.

[10] Coughlin SS (2012). Anxiety and depression: linkages with viral diseases. Public Health Rev.

[11] Brooks SK, Webster RK, Smith LE (2020). The psychological impact of quarantine and how to reduce it: rapid review of the evidence. Lancet.

[12] Reger MA, Stanley IH, Joiner TE (2020). Suicide mortality and coronavirus disease 2019—a perfect storm?. JAMA Psychiatry.

[13] Schneider D, Harknett K, McLanahan S (2016). Intimate partner violence in the Great Recession. Demography.

[14] Millett L, Lanier P, Drake B (2011). Are economic trends associated with child maltreatment? Preliminary results from the recent recession using state level data. Child Youth Serv Rev.

[15] Brooks-Gunn J, Schneider W, Waldfogel J (2013). The Great Recession and the risk for child maltreatment. Child Abuse Negl.

[16] Seery MD, Holman EA, Silver RC (2010). Whatever does not kill us: cumulative lifetime adversity, vulnerability, and resilience. J Pers Soc Psychol.

[17] Seery MD, Leo RJ, Lupien SP, Kondrak CL, Almonte JL (2013). An upside to adversity? Moderate cumulative lifetime adversity is associated with resilient responses in the face of controlled stressors. Psychol Sci.

[18] Seery MD (2011). Resilience: a silver lining to experiencing adverse life events?. Curr Dir Psychol Sci.

